# High-grade Non-small Cell Neuroendocrine Carcinoma of the Esophagus

**DOI:** 10.7759/cureus.2416

**Published:** 2018-04-03

**Authors:** Stephen J Hjerpe, Umar Rahim, Muhammad Shariq Usman, Amna Ansari, Waliul Chowdhury, Muhammad Uzair Lodhi, Mustafa Rahim

**Affiliations:** 1 Department of Medicine, Lincoln Memorial University-Debusk College of Osteopathic Medicine; 2 Pre-Medical Student, Department of Sciences, Queens University of Charlotte, Nc; 3 Internal Medicine, Civil Hospital Karachi, Dow University of Health Sciences, Karachi, Pakistan; 4 Medicine, Mcmaster University Michael G. Degroote School of Medicine, Canada; 5 Medical Student, Department of Medicine, Raleigh General Hospital, Beckley, Wv; 6 Assistant Clinical Professor of Internal Medicine, West Virginia University School of Medicine

**Keywords:** neuroendocrine carcinoma, dysphagia, esophagogastroduodenoscopy, ki-67 index, lactate dehydrogenase, somatostatin receptor imaging, positron emission tomography, cisplatin, carboplatin

## Abstract

High-grade neuroendocrine carcinoma (NEC) of the esophagus is an extremely aggressive and rare disease, which is still not well understood. In this case report, we discuss a 73-year-old male patient that presented with the sole complaint of dysphagia to solid foods. During our evaluation of the patient, a six-centimeter esophageal mass was found on esophagogastroduodenoscopy (EGD). A diagnosis of poorly differentiated (high-grade) non-small cell neuroendocrine carcinoma was made after a histological analysis and immunostaining. We attempted to highlight the diagnosis, evaluation process, and treatment options related to this entity. Our review of the literature revealed that further research is needed, focusing on neuroendocrine carcinomas of the esophagus and how this entity differs from some of the more well-known neuroendocrine neoplasms in terms of management.

## Introduction

Neuroendocrine carcinoma of the esophagus is classified within the gastroenteropancreatic neuroendocrine neoplasms (GEP-NENs) based on the 2010 World Health Organization (WHO) classification system. GEP-NENs are divided into three grades based on the Ki-67 index, which is a measurement of the proliferative content displayed by neoplastic cells. A Ki-67 index greater than 20% is required to be considered a grade 3 or neuroendocrine carcinoma (NEC) [1]. NECs are further classified into small cell, which is more common, and large cell [2]. The incidence rate of GEP-NENs was found to be 3.65 per 100,000 individuals every year, out of which 1.3% are located within the esophagus [1-3]. These statistics help illustrate the rare occurrence of the entity discussed in this case report, compared to other neuroendocrine neoplasms within the general population. NECs are also known for being extremely aggressive, as they are usually metastasized by the time they are diagnosed [4]. Patients with high-grade NECs affecting the gastrointestinal system are found to have a median survival time of one month without intervention and 11 months if chemotherapy is administered [5].

## Case presentation

A 73-year-old Caucasian male patient presented to the office with a complaint of dysphagia only to solid foods lasting three to four weeks. The patient's past medical history was significant for gastroesophageal reflux disease (GERD), which was treated with protonix. The patient's history included a 55-pack-year cigarette use, occasional alcohol use, and no recreational drug use. Family history was noncontributory. On physical examination, the patient was found to have a supple neck without thyromegaly, masses, or lymphadenopathy. The patient did exhibit bilateral carotid bruits, a soft systolic murmur loudest at the right sternal border, and decreased peripheral pulses. He had a soft, nontender abdomen without hepatosplenomegaly, and bowel sounds were present. Laboratory workup was normal.

**Figure 1:**
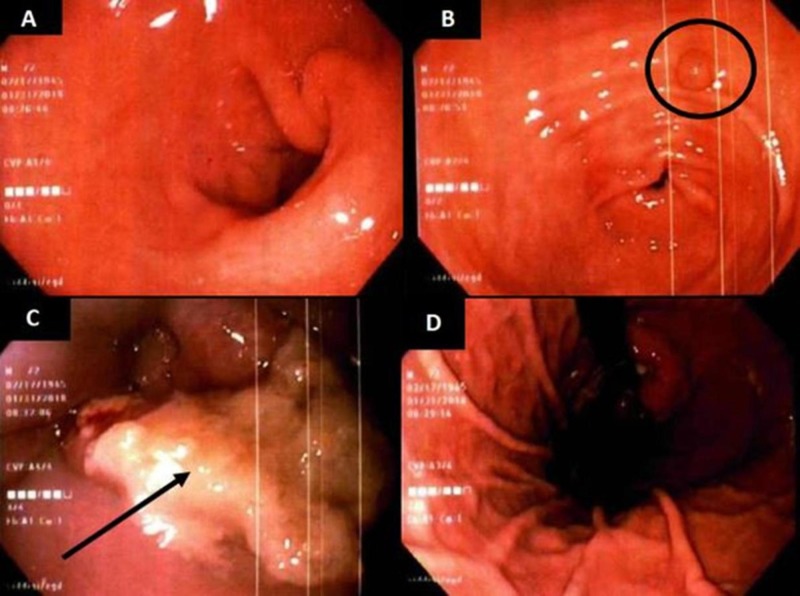
EGD imaging of the duodenum (A), antrum with gastric polyp shown with circle (B), distal esophageal mass with ulceration shown with arrow (C), and fundal view (D). EGD: esophagogastroduodenoscopy

Given the patient’s initial presentation, age, history of smoking, and GERD, a barium swallow was performed, which was within normal limits. We followed with an esophagogastroduodenoscopy (EGD), as shown in Figure 1, during which several biopsies were taken. An esophageal mass was found in the distal esophagus, between 36-42 centimeters from the incisor line, which appeared to extend through the Z-line, which measured 41 centimeters. The esophageal mass biopsies were taken at the proximal and distal ends of the lesion.

**Figure 2:**
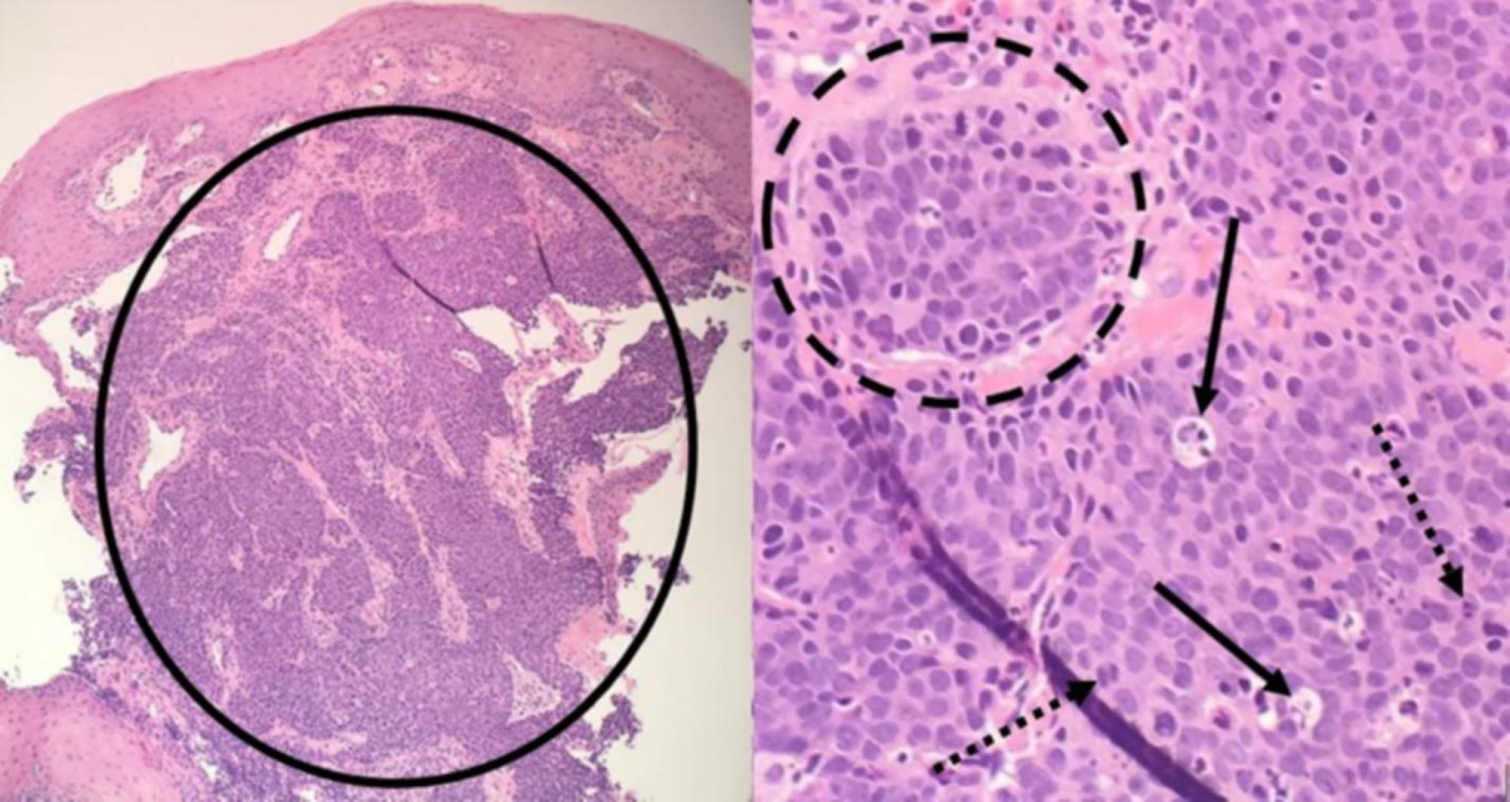
Biopsy specimen from the proximal esophageal mass (Hematoxylin and Eosin stain) showing basophilic cells with variable size and shape representing the tumor cells in an organoid pattern (solid circle) beneath a layer of non-keratinized, stratified squamous epithelium. The tumor cells have hyperchromatic nuclei and arrange into nests (dashed circle). Mitotic figures (dashed arrows) and apoptotic bodies (solid arrows) are found throughout.

A histological analysis of the specimens showed no Helicobacter pylori, antral-type gastric mucosa with minimal chronic gastritis from the antrum biopsy, epithelial hyperplasia with expanding lamina propria, indicative of a hyperplastic polyp from the gastric biopsy, necrotic tissue with small, atypical, cellular nests at the distal mass biopsy, and a poorly differentiated carcinoma from the proximal end of the esophageal mass, as shown in Figure 2.

**Figure 3:**
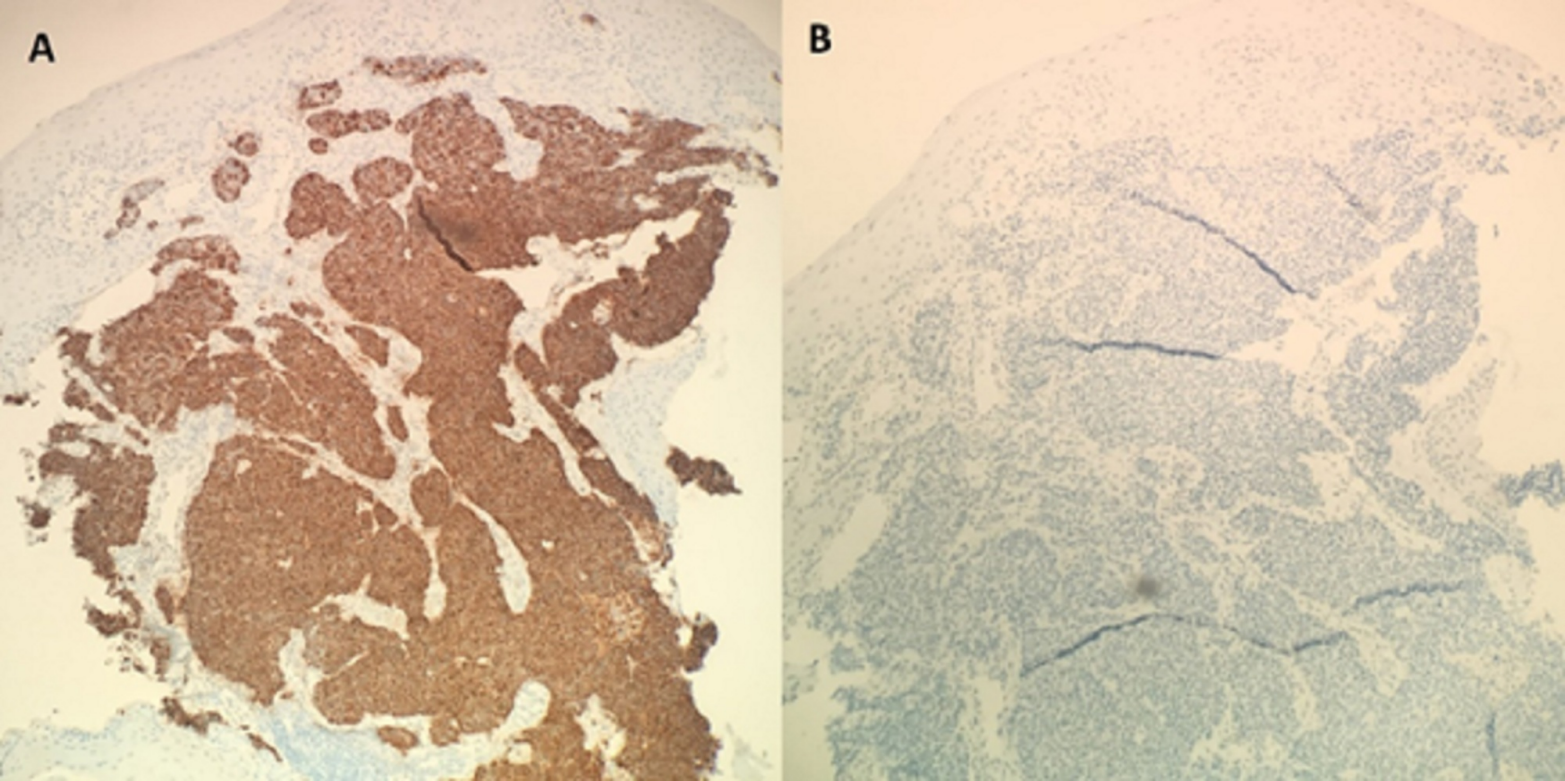
Histological comparison of positive immunostaining with chromogranin (A) and negative immunostaining with TTF-1 (B).

The proximal esophageal biopsy was sent to Mayo Clinic for further analysis with immunostaining. The tumor cells were found to be positive for keratin AE1/AE3, chromogranin, synaptophysin, CDX2, weakly positive for Islet-1, but negative for p40, keratin 5/6, and thyroid transcription factor (TTF-1), as shown in Figure 3. Ki-67 quantitative imaging analysis showed a 90% labeling index for the tumor cells. The morphologic features and staining pattern supported the diagnosis of poorly differentiated (high-grade) non-small cell neuroendocrine carcinoma. Luminal budding yeast and pseudohyphae were also found on the specimen, consistent with Candida species.

## Discussion

GEP-NENs are most commonly found in males between the ages of 50 and 70 years [1,6-9]. While there is limited information about the risk factors for developing an esophageal NEC, a literature review by Ilett et al. [4] found a correlation between smoking and alcohol consumption in this patient population. Our patient has a significant history of tobacco smoking, leading us to consider this as a possible contributing factor for disease progression. Dysphagia appears to be the most common presenting complaint experienced by individuals diagnosed with esophageal NECs [9], and it was the primary symptom found in our patient. While our patient did not admit to weight loss at the initial evaluation, a detailed review of medical records showed a 21-pound loss over the year leading up to diagnosis. During our literature review, we were unable to determine if small versus large cell carcinoma had any role in the prognostic outcome for patients with esophageal NEC due to inconclusive data. However, an elevated lactate dehydrogenase (LDH) and platelet counts are strong factors in predicating poor survival from gastrointestinal NECs [5]. The exact mechanism is still not well understood, but these labs are believed to be associated with aggressive tumor types and an increased inflammatory response [5]. In our patient, the platelet count was found to be 177 K/uL and LDH at 191 IU/L, both within normal limits.

**Figure 4:**
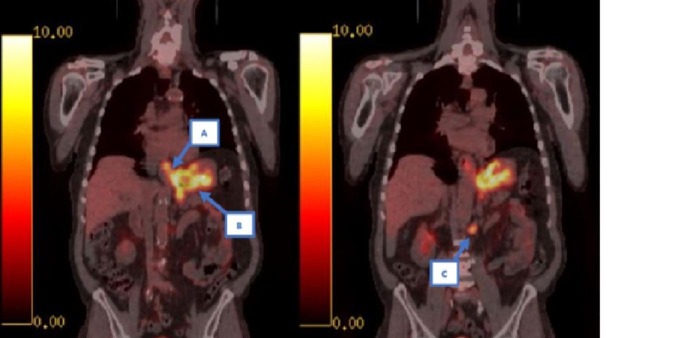
PET coronal views showing hypermetabolic areas of the distal esophagus/fundus (A), lesser curvature (B), and adjacent to the abdominal aorta at the level of the left renal vein (C). PET: positron emission tomography

**Figure 5:**
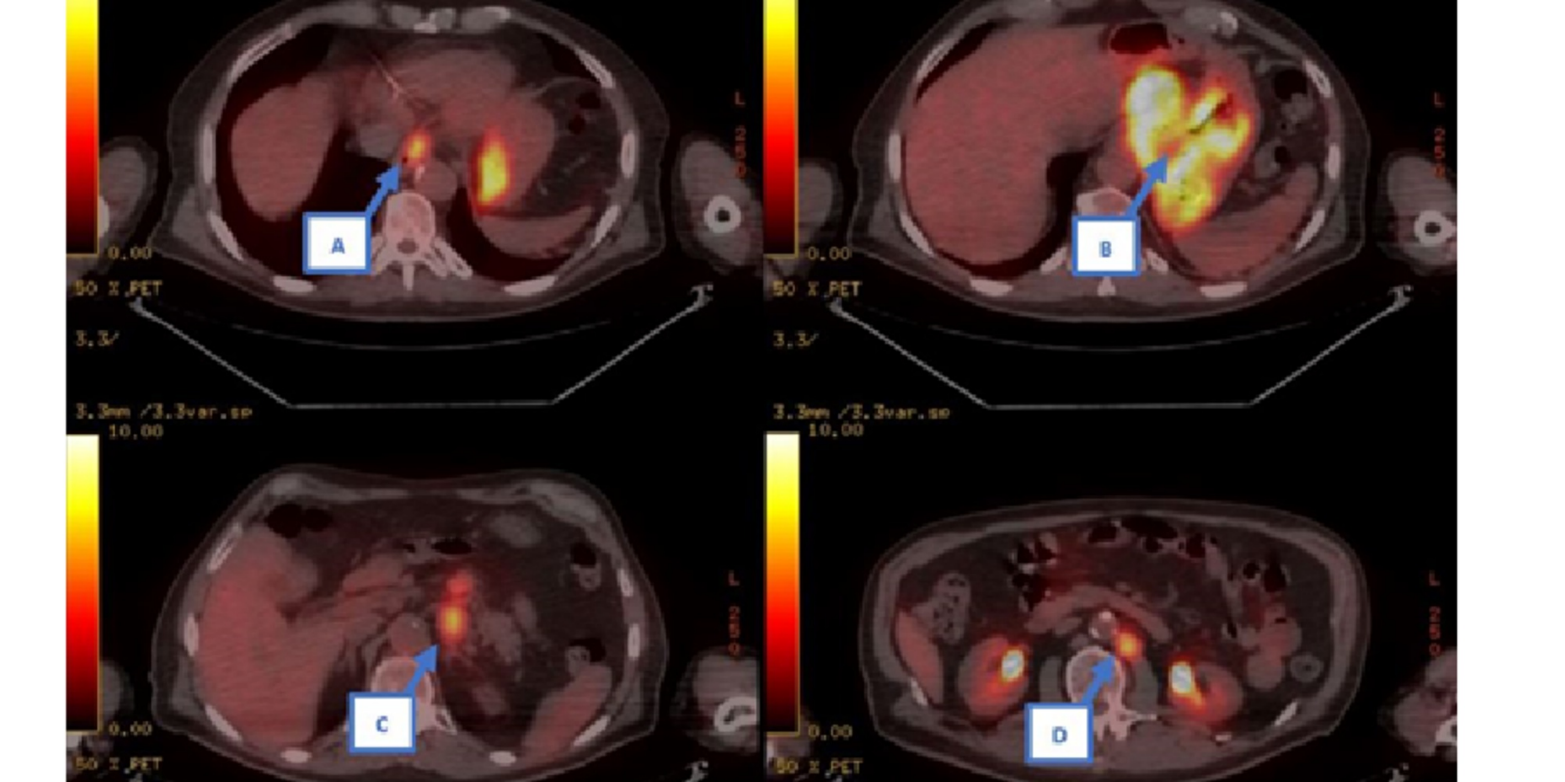
PET axial views showing hypermetabolic areas of the distal esophagus (A), stomach (B), retrogastric area (C), and adjacent to abdominal aorta at the level of left renal vein (D). PET: positron emission tomography

After a GEP-NEN diagnosis is established, it is recommended that patients should be evaluated with somatostatin receptor imaging (SRI) or positron emission tomography (PET) prior to developing a treatment plan [1]. In our patient, F-18 labeled FDG PET imaging was performed, as shown in Figures 4-5. There was increased activity at the distal esophagus and gastric fundus in addition to at least three nodal metastases located along the lesser curvature of the stomach, the retrogastric area, and adjacent to the abdominal aorta at the left renal vein. In addition, magnetic resonance imaging (MRI) of the brain was performed with no metastasis seen.

With no established standard of care, clinicians currently face the task of treating patients with esophageal NECs with a lot of uncertainty and limited information. Current treatment regimens are being based on research from more well-known NECs originating from other areas of the body, such as the lungs [5]. While surgery seems to increase the survival time of patients, it is generally only used in patients with limited disease [4,6]. In our patient, surgery was unlikely due to the high grade and metastasis at the time of diagnosis, so further nonsurgical options were explored. A combination of chemotherapeutic medication, such as cisplatin, carboplatin, etoposide, and irinotecan, are the most commonly agreed-upon treatment options. A correlation between medication response rate and the Ki-67 index has been observed, with Ki-67 rates greater than 55% being more responsive to platinum-based medications, such as cisplatin and carboplatin [5]. Our patient exhibited a Ki-67 index of 90% supporting the classification of a high-grade NEC (as previously mentioned) and directing us to consider a treatment plan consisting of cisplatin or carboplatin.

## Conclusions

The literature is lacking on the management of patients with neuroendocrine carcinoma of the esophagus due to the low incidence rate of this disease. In this case report, we attempted to present our patient and evaluate any correlations within the literature to provide a further understanding of disease progression and how, as health care providers, we can treat such an aggressive neoplasm with poor prognostic outcomes. The majority of the conclusions found within the literature on esophageal NECs are being based on retrospective data analysis, and it is evident that further research focusing on early detection and treatment regimens is needed, in order to increase the survival rates of patients facing this grim diagnosis.
